# MYC deregulates TET1 and TET2 expression to control global DNA (hydroxy)methylation and gene expression to maintain a neoplastic phenotype in T-ALL

**DOI:** 10.1186/s13072-019-0278-5

**Published:** 2019-07-02

**Authors:** Candace J. Poole, Atul Lodh, Jeong-Hyeon Choi, Jan van Riggelen

**Affiliations:** 1 Department of Biochemistry and Molecular Biology, Augusta University, 1410 Laney-Walker Blvd., Augusta, GA 30912 USA; 2 Georgia Cancer Center, Augusta University, 1410 Laney-Walker Blvd., Augusta, GA 30912 USA

**Keywords:** MYC, TET1, TET2, DNA methylation, DNA hydroxymethylation, Leukemia/lymphoma

## Abstract

**Background:**

While aberrant DNA methylation is a characteristic feature of tumor cells, our knowledge of how these DNA methylation patterns are established and maintained is limited. DNA methyltransferases and ten-eleven translocation methylcytosine dioxygenases (TETs) function has been found altered in a variety of cancer types.

**Results:**

Here, we report that in T cell acute lymphoblastic leukemia (T-ALL) the *MYC* oncogene controls the expression of *TET1* and *TET2* to maintain 5-methylcytosine (5mC) and 5-hydroxymethylcytosine (5hmC) patterns, which is associated with tumor cell-specific gene expression. We found that cellular senescence and tumor regression upon MYC inactivation in T-ALL was associated with genome-wide changes in 5mC and 5hmC patterns. Correlating with the changes in DNA (hydroxy)methylation, we found that T-ALL overexpress *TET1*, while suppressing *TET2* in a MYC-dependent fashion. Consequently, MYC inactivation led to an inverse expression pattern, decreasing *TET1*, while increasing *TET2* levels. Knockdown of *TET1* or ectopic expression of *TET2* in T-ALL was associated with genome-wide changes in 5mC and 5hmC enrichment and decreased cell proliferation, suggesting a tumor promoting function of TET1, and a tumor suppressing role for TET2. Among the genes and pathways controlled by TET1, we found ribosomal biogenesis and translational control of protein synthesis highly enriched.

**Conclusions:**

Our finding that MYC directly deregulates the expression of *TET1* and *TET2* in T-ALL provides novel evidence that MYC controls DNA (hydroxy)methylation in a genome-wide fashion. It reveals a coordinated interplay between the components of the DNA (de)methylating machinery that contribute to MYC-driven tumor maintenance, highlighting the potential of specific TET enzymes for therapeutic strategies.

**Electronic supplementary material:**

The online version of this article (10.1186/s13072-019-0278-5) contains supplementary material, which is available to authorized users.

## Background

The MYC oncogene is involved in the pathogenesis of 60–70% of all human cancers, including T cell acute lymphoblastic leukemia (T-ALL) (reviewed in [1, 2]). MYC encodes for a transcription factor that controls the expression of a large number of genes, thereby deregulating a variety of cellular processes, ultimately leading to autonomous cell proliferation, growth, and angiogenesis, while blocking cellular differentiation (reviewed in [3, 4]). Its wide implications in human tumorigenesis and the notion that tumors can be dependent on enhanced MYC expression, exhibiting oncogene addiction, make the oncogene and its network a highly promising target for therapeutic strategies [5–7].

While the many functions of MYC were first attributed to its ability to both activate and repress transcription of a large number of genes through direct binding to their promoters, more and more reports challenge this dogma. In the classic model, MYC, heterodimerized with MAX, recognizes a DNA-motif named E-box (CACGTG), thereby transactivating canonical target genes through recruitment of chromatin-modifying cofactors [8, 9]. At the same time, through interactions with other transcription factors such as MIZ-1, the transactivating function of MYC-MAX can be fine-tuned, or even inverted to repress the transcription of genes harboring an INR DNA-motif (reviewed in [10]). Furthermore, MYC has been demonstrated to bind virtually all active promoters and many enhancers, thereby boosting the transcriptional output of a given cell through various mechanisms [11–14]. In parallel, it has been emerging that MYC regulates chromatin structure in a genome-wide fashion [15–18], suggesting a mechanism that extends beyond the classic model of a site-specific transcription factor (reviewed in [19]).

We and others have demonstrated that cellular senescence accompanied by extensive chromatin remodeling is an important mechanism of tumor regression upon MYC inactivation in T-ALL, and other cancer types [16, 20, 21]. During this process, broad changes occur in histone methylation (increase of H3K9me3) and acetylation (decrease of H4ac), suggesting that during tumor maintenance MYC maintains large areas of active chromatin. Consequently, MYC inactivation is associated with genome-wide changes in the epigenetic landscape. Indeed, there is growing evidence that MYC induces genome-wide alterations in chromatin in order to elicit its neoplastic properties (reviewed in [19]). As first evidence, N-MYC transcriptional upregulation of the histone acetyltransferase, *GCN5*, was reported to cause genome-wide acetylation of histones [15]. Similarly, we reported that MYC inactivation in T-ALL triggers genome-wide changes in histone acetylation and methylation associated with cellular senescence and tumor regression [16]. Furthermore, MYC recently has been shown to suppress chromatin regulators, *SIN3B*, *HBP1*, *SUV420h1*, and *BTG* via the miR-17-92 cluster [17]. Together, these results indicate that MYC controls genome-wide chromatin domains through modulating the expression of chromatin-modifying enzymes in order to create an epigenetic landscape that favors neoplastic gene expression programs.

Despite the recent reports teasing out the function of MYC as global regulator of transcription, it remains elusive how MYC establishes and maintains DNA methylation as an important component of chromatin structure. Tumor cells typically display global hypomethylation of repetitive DNA elements which contributes to genomic instability, while promoter and CpG island hypermethylation extinguish transcription of tumor suppressor genes. DNA methylation as 5-methylcytosine (5mC) is established by de novo DNA methyltransferases (DNMTs), DNMT3A and DNMT3B, while DNMT1 preferentially binds hemi-methylated DNA and maintains methylation to prevent passive demethylation (reviewed in [22]). Aberrant DNA methylation is a characteristic feature of tumor cells and is known to contribute to tumorigenesis in human neoplasia [23–25]. Shedding light on how MYC controls DNA methylation in T-ALL and Burkitt lymphoma, we recently reported that MYC causes the overexpression of *DNMT3B*, maintaining specific 5mC and thus gene expression patterns which are important for tumor maintenance [26]. However, the role of ten-eleven translocation methylcytosine dioxygenases (TETs) in this context remained elusive.

TET enzymes convert 5mC to 5-hydroxymethylcytosine (5hmC) and other cytosine intermediates (5-formylcytosine (5fC) and 5-carboxycytosine (5caC)), which contribute to the process of active DNA demethylation through base excision repair processes. Opposite to the repressive effects 5mC often has at promoters, the enrichment of 5hmC at gene promoters correlates with increased gene expression [27–30]. It has been reported that genome-wide distribution of 5hmC is overall reduced in neoplastic tissue and tumor cell-specific 5hmC occurs at specific gene coding regions, revealing the importance of 5hmC in modulating gene expression [27, 31]. However, much remains elusive how 5mC and 5hmC patterns contribute to the deregulation of gene expression during MYC-driven tumorigenesis and tumor maintenance.

Understanding the molecular mechanisms how tumor cell-specific DNA (hydroxy)methylation patterns are established and maintained by MYC may provide novel therapeutic strategies, aiming at specific components of the DNA (de)methylating machinery. Here, we report that in T-ALL, the MYC oncogene controls the expression of both *TET1* and *TET2*, which in turn contribute to tumor cell-specific 5mC and 5hmC patterns in a genome-wide fashion with importance for tumor maintenance.

## Results

### Tumor regression upon MYC inactivation in T-ALL is associated with genome-wide changes in DNA (hydroxy)methylation patterns

Inactivation of the MYC oncogene in a mouse model of T-ALL (*EµSRα*-*tTAα;tet*-*o*-*MYC*) causes sustained tumor regression by eliciting the phenomenon of oncogene addiction [5]. Using this T-ALL model, we have previously demonstrated that tumor regression upon MYC inactivation depends on activation of cellular senescence pathways associated with genome-wide changes in chromatin structure including histone acetylation (decrease of H4ac) and methylation (increase of H3K9me3), associated with heterochromatin formation [16, 21, 32]. Furthermore, we recently reported that MYC deregulates the expression of methylation modifiers, *DNMT3B* and *DNMT1*, essential for tumor maintenance [26]. Together, these findings suggest that during tumor maintenance MYC maintains large domains of active chromatin, and that tumor regression upon MYC inactivation is tightly linked to changes in expression of chromatin modifiers resulting in genome-wide changes to chromatin structure.

**Fig. 1:**
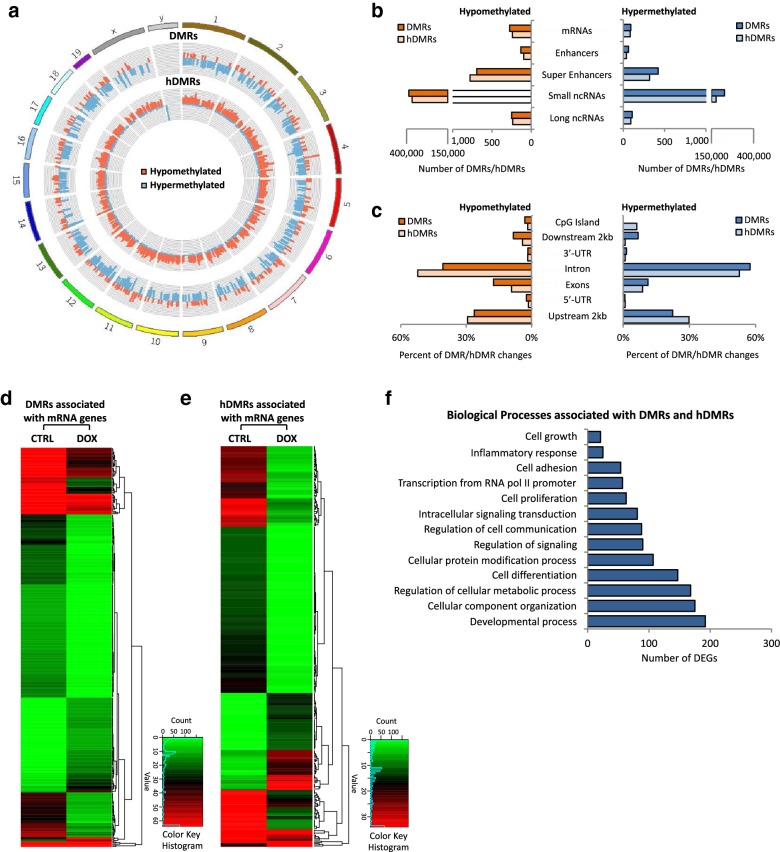
Tumor regression upon MYC inactivation in T-ALL is associated with genome-wide changes in DNA (hydroxy)methylation. MeDIP- and hMeDIP-seq analysis of T-ALL cells (6780) derived from *EµSRα*-*tTAα;tet*-*o*-*MYC* mice before and upon MYC inactivation through treatment with 20 ng/mL DOX for 2 days. **a** Genomic distribution of DMRs and hDMRs is displayed as chromosome-based circular plot. Cutoff: log2FC ≥ 1 with a *P* value of ≤ 10^−4^. **b** Hypo- or hypermethylated DMRs and hDMRs are shown annotated for their association with mRNAs, enhancers, super-enhancers, small noncoding RNAs, and long noncoding RNAs. **c** Hypo- or hypermethylated DMRs and hDMRs associated with mRNAs are shown annotated for *cis*-regulatory elements: CpG islands, exons, introns, 5′-/3′-UTRs, and sequences 2 kbp upstream or downstream of the nearest gene. Heatmap showing hierarchical clustering of **d** DMRs and **e** hDMRs associated with protein-coding genes. Gene names are listed in Additional file 3. **f** Gene ontology analysis (DAVID) indicating biological processes associated with DMRs and hDMRs

To determine the effect MYC inactivation in tumors has on DNA methylation (5mC) and hydroxymethylation (5hmC), we carried out methylated DNA immunoprecipitation (MeDIP- and hMeDIP-seq, respectively) analysis, taking advantage of the tetracycline-regulated *c*-*myc* allele in T-ALL cells derived from *EµSRα*-*tTAα;tet*-*o*-*MYC* mice (Fig. 1). We compared mouse T-ALL cells (6780) in vitro before (CTRL) and upon inactivation of MYC by adding 20 ng/mL doxycycline (+DOX) to the culture medium for 2 days. *MYC* inactivation was validated by RT-qPCR (Additional file 1: Fig. S1). For each sample, 45–60 million Illumina sequencing reads were generated. Of these, ~ 45–80% were successfully mapped to either strand of the mouse genome (mm10). To identify significantly differentially methylated regions (DMRs) and differentially hydroxymethylated regions (hDMRs), we performed a genome-wide, unbiased DMR and hDMR detection using a complete tiling of the mouse genome using a cutoff of log2FC ≥ 1 with a *P* value of ≤ 10^−4^.

We identified a total of 615,875 DMRs and 545,504 hDMRs that become significantly hypo- or hypermethylated upon MYC inactivation for 2 days. The genomic location of DMRs and hDMRs between MYC on and off states, displayed as circular plot, indicates genome-wide changes in 5mC and 5hmC distribution (Fig. 1a). We next mapped the DMRs and hDMRs (both hypo- and hypermethylated) to the annotated RefSeq genes in the mouse genome. We found 366 and 323 DMRs and hDMRs associated with mRNAs, 186 and 123 with enhancers, 1113 and 1093 with super-enhancers, 613,839 and 543,625 with small noncoding RNAs (ncRNAs), and 358 and 326 with long ncRNAs, respectively (Fig. 1b). We further annotated DMRs and hDMRs associated with protein-coding genes for *cis*-regulatory elements. Of the those DMRs and hDMRs, we found 3.11% and 1.78% associated with CpG islands, 17.46% and 9.20% with exons, 40.67% and 52.23% introns, 4.07% and with 3.26% 5′- or 3′-UTRs, and 34.69% and 33.53% with sequences 2 kbp or more upstream or downstream of the nearest gene, respectively (Fig. 1c). The fold change of DMRs and hDMRs associated with protein coding genes is displayed as heatmap, respectively (Fig. 1d, e). Of the 366 and 323 genes associated with DMRs and hDMRs, we found 275 and 240 to increase, and 91 and 83 to decrease in (hydroxy)methylation, respectively.

To determine the biological processes associated with DNA methylation changes upon MYC inactivation in T-ALL, we performed gene ontology analysis using the Database for Annotation, Visualization and Integrated Discovery (DAVID). Consistent with MYC’s broad reach, we found a wide variety of processes associated with DMRs and hDMRs, ranging from regulation of cell growth and proliferation, to differentiation and metabolism (Fig. 1f and Additional file 3). Taken together, the MeDIP- and hMeDIP-seq analyses reveal genome-wide changes in 5mC and 5hmC distribution associated with a wide variety of biological processes upon MYC inactivation, indicating that MYC maintains tumor cell-specific DNA (hydroxy)methylation patterns in T-ALL.

### *TET1* and *TET2* expression levels in T-ALL are MYC-dependent and are inversed upon MYC inactivation

**Fig. 2:**
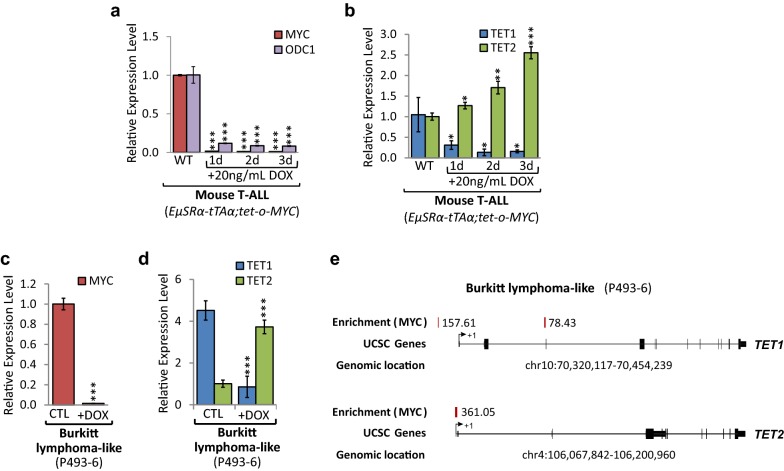
*TET1* and *TET2* levels are dependent on MYC expression. MYC inactivation in T-ALL cells (6780) derived from *EµSRα*-*tTAα;tet*-*o*-*MYC* mice, and human Burkitt lymphoma-like (P493-6) cells, harboring a tetracycline-regulated *c*-*MYC* allele, in a time-dependent manner for 1, 2, and 3 days using 20 ng/mL DOX. Mouse T-ALL cells: **a** RT-qPCR analysis of *MYC* and its canonical target gene *Ornithine Decarboxylase 1* (*ODC1*), and **b** of *TET1* and *TET2*. RT-qPCR data were normalized to *UBC*. Human Burkitt lymphoma-like cells: **c** RT-qPCR of *MYC* and **c** *TET1* and *TET2* in P493-6 cells before (CTL) and upon MYC inactivation through treatment with 20 ng/mL DOX for 2 days (+DOX). RT-qPCR data were normalized to *RPL13A*. **d** MYC ChIP-seq data for P493-6 cells obtained from Sabo et al. [13] indicating enrichment score for MYC at the *TET1* and *TET2* loci. Traces were generated based on reference genome hg19 using the UCSC Genome Browser. The chromosomal location is indicated in bp. MYC binding peaks are displayed as red vertical bars; numbers represent the relative fold enrichment for MYC. Exons are displayed as black vertical bars, the UTR is represented by a black line, and the transcription start site (TSS) is marked by an arrow indicating the direction of transcription

We previously reported that MYC causes the overexpression of *DNMT1* and *DNMT3B* in T-ALL, thereby establishing and maintaining specific 5mC and thus gene expression patterns [26]. To further investigate the mechanism underlying global 5mC and 5hmC changes upon MYC inactivation, we performed gene expression profiling for TET enzymes (Fig. 2). We compared T-ALL cells (6780) derived from *EµSRα*-*tTAα;tet*-*o*-*MYC* mice, harboring a tetracycline-regulated c-myc allele, before (CTRL) and upon MYC inactivation (+DOX) over the course of 3 days. RT-qPCR analysis for *MYC* and its canonical target gene, *Ornithine Decarboxylase 1* (*ODC1*), verified MYC inactivation (96.17-fold, *P* = 8.41 × 10^−9^, and 12.36-fold, *P* = 1.2 × 10^−5^ lower on day 3, respectively) in *EµSRα*-*tTAα;tet*-*o*-*MYC*-derived T-ALL cells (Fig. 2a). Subsequently, RT-qPCR for *TET1* and *TET2* revealed a direct correlation between *MYC* and *TET1* expression levels, while showing an inverse correlation between *MYC* and *TET2* levels (Fig. 2b). In mouse T-ALL, *TET1* mRNA levels decreased significantly (6.63-fold, *P* = 0.021), while *TET2* mRNA levels increased significantly (2.55-fold, *P* = 9.4 × 10^−5^) upon MYC inactivation after 3 days.

Furthermore, to validate our results in mouse T-ALL we used P493-6 cells as model for high and low MYC expression in human lymphocytes, allowing for MYC inactivation. We compared human Burkitt lymphoma-like cells (P493-6), harboring a tetracycline-regulated c-myc allele, before (CTL) and upon MYC inactivation (+DOX) over the course of 2 days. RT-qPCR showed a significant decrease (64.39-fold, *P* = 7.67 × 10^−6^) in *MYC* mRNA expression upon 2 days of DOX treatment (Fig. 2c). Mimicking our results in T-ALL, *TET1* mRNA decreased (5.25-fold, *P* = 0.0015), while *TET2* levels increased (3.72-fold, *P* = 2.20 × 10^−5^) (Fig. 2d). Together, these results indicate that *TET1* expression is high, while *TET2* is low in T-ALL derived from *EµSRα*-*tTAα;tet*-*o*-*MYC* and in human Burkitt lymphoma, revealing a direct correlation between *MYC* and *TET1*, and an inverse correlation between *MYC* and *TET2* expression levels.

To further determine whether MYC directly binds to the *TET1* and *TET2* genes and controls their transcription, we analyzed chromatin immunoprecipitation (ChIP-seq) data for P493-6 cells generated by Sabo et al. [13] (see Fig. 2e). We found MYC binding to the genomic *TET1* and *TET2* loci to be significantly enriched in P493-6 cells, revealing that MYC occupies sequences at the *TET1* locus upstream of exon 1 (enrichment 157.61) and exon 3 (enrichment 78.43), as well as the *TET2* locus downstream of exon 1 (enrichment 361.05). We conclude that high *TET1* and low *TET2* expression levels in mouse T-ALL and human Burkitt lymphoma-like cells are directly MYC-dependent and are inversed upon MYC inactivation.

### *TET1* is overexpressed, while *TET2* is suppressed in human T-ALL cell lines and clinical specimens

**Fig. 3:**
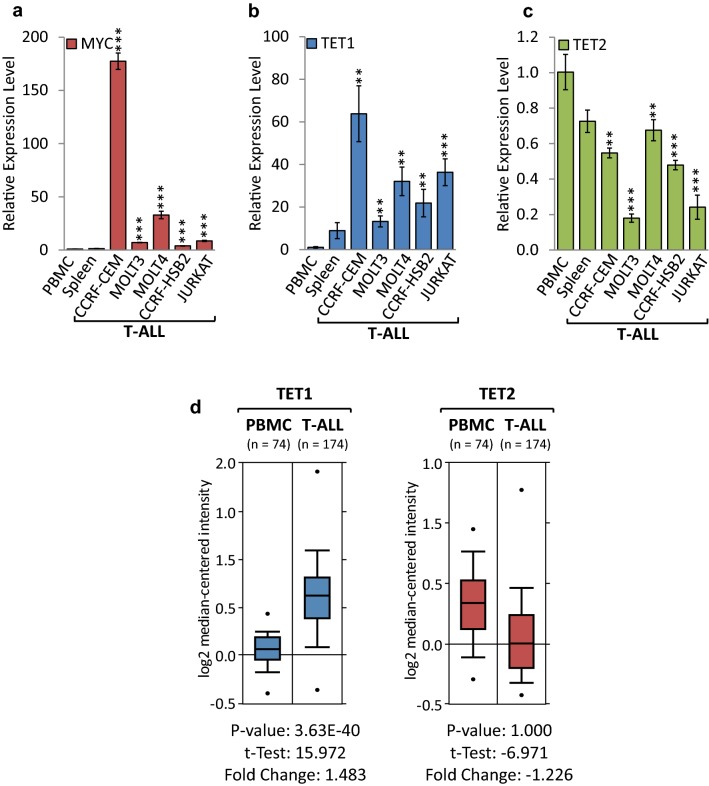
*TET1* is overexpressed, while *TET2* is suppressed in human T-ALL cell lines and clinical specimens. Expression profiling of *TET1* and *TET2* in human T-ALL cell lines and clinical specimens compared to non-malignant tissue. RT-qPCR of **a** *MYC*, **b** *TET1* and **c** *TET2* in human T-ALL (CCRF-CEM, MOLT3, MOLT4, CCRF-HSB2, JURKAT) compared to peripheral blood mononuclear cells (PBMCs) and spleen obtained from healthy donors. RT-qPCR data were normalized to *RPL13A*. Error bars represent mean ± SEM; *n* = 3; two-tailed Student’s *t* test: *P < 0.05; **P < 0.01; ***P < 0.001. **d** *TET1* and *TET2* expression profiles in clinical specimens obtained though Oncomine (http://www.oncomine.org) from Haferlach et al. [33]. *TET1* and *TET2* mRNA levels are displayed for T-ALL (*n* = 174) compared to PBMCs (*n* = 74). Boxes indicate the interquartile range; the line within the box represents the median. Whiskers indicate the non-outlier minimum and maximum. Outliers are represented by circles. Significant *P* values and fold changes are indicated

To translate our results from transgenic models to patient-derived cell lines overexpressing endogenous MYC, we performed gene expression profiling for *TET1* and *TET2*, comparing a panel of human T-ALL cell lines to peripheral blood mononuclear cells (PBMCs) and spleen obtained from healthy donors (Fig. 3a–c). RT-qPCR analysis indicated that *MYC* mRNA levels were significantly higher in CCRF-CEM (177.43-fold), MOLT3 (7.04-fold), MOLT4 (32.93-fold), CCRF-HSB2 (3.89-fold), and JURKAT (8.08-fold), compared to normal PBMCs. RT-qPCR analysis indicated that *TET1* mRNA levels were significantly higher in CCRF-CEM (63.80-fold), MOLT3 (13.23-fold), MOLT4 (32.07-fold), CCRF-HSB2 (21.83-fold), and JURKAT (36.34-fold), compared to normal PBMCs. In contrast, we found *TET2* expression to be significantly lower in CCRF-CEM (1.83-fold), MOLT3 (5.56-fold), MOLT4 (1.48-fold), CCRF-HSB2 (2.09-fold), and JURKAT (4.14-fold). To test whether clinical T-ALL specimens resemble our results from established cell lines, we analyzed publically available expression data obtained from Haferlach et al. [33] via Oncomine (Fig. 3d). We found that *TET1* mRNA expression levels are elevated (1.483-fold, *P *= 3.63 × 10^−40^), while *TET2* expression levels are lower (1.226-fold, *P *= 1.0) in clinical T-ALL (*n* = 174) than in normal PBMCs (*n* = 74), which is consistent with our findings in cell lines. Taken together, the expression profiling reveals that *TET1* is overexpressed, while *TET2* is suppressed in patient-derived T-ALL cell lines and clinical specimens, mimicking the above results from transgenic models.

### *TET1* knockdown reduces cell proliferation of human T-ALL cells

**Fig. 4:**
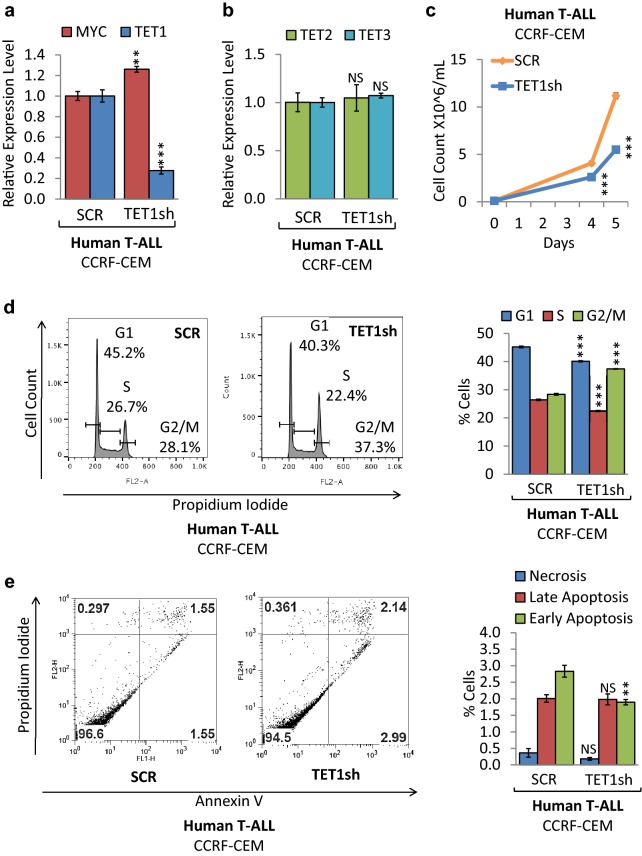
*TET1* KD reduces cell proliferation of human T-ALL. Human T-ALL cells (CCRF-CEM) were compared before (SCR) and upon *TET1* KD (TET1sh). RT-qPCR of **a** *MYC* and *TET1*, **b** *TET2* and *TET3*. RT-qPCR data were normalized to *RPL13a*. **c** Growth curve comparing viable cell counts. **d** Flow cytometric cell cycle analysis based on propidium iodide (PI) staining. The cell cycle distribution (G1, S, and G2/M) is displayed in percent. **e** Flow cytometric analysis of apoptosis based on Annexin V/PI staining. Flow cytometry profile of Annexin V staining (*X* axis) and PI (*Y* axis) is shown for representative samples. The lower right quadrant indicates the percentage of early apoptotic cells in each condition; the upper right quadrant indicates the percentage of late apoptotic cells; the upper left quadrant indicates the percentage of necrotic cells. Apoptotic cells (Annexin V-positive cells) are displayed as the percentage of gated cells. Error bars represent mean ± SEM; *n* = 3; two-tailed Student’s *t* test: *P < 0.05; **P < 0.01; ***P < 0.001

To determine whether loss of TET1 function affects tumor cell proliferation and viability, we carried out shRNA-mediated *TET1* knockdown (KD) in human T-ALL cells (CCRF-CEM). CCRF-CEM is an established T-ALL cell line, exhibiting high MYC expression. We compared CCRF-CEM cells upon shRNA-mediated knockdown of *TET1* to a shRNA control (Fig. 4). First, we validated *TET1* KD as well as *MYC*, *TET2*, and *TET3* expression levels by RT-qPCR (Fig. 4a, b). Compared to scrambled (SCR) control, CCRF-CEM cells expressing TET1sh show 3.63-fold less *TET1* mRNA levels, while *MYC*, *TET2*, and *TET3* levels were similar. The corresponding growth curve indicates that *TET1* KD in CCRF-CEM decreased cell proliferation (Fig. 4c). To determine a mechanism underlying reduced cell proliferation, flow cytometric cell viability and cell cycle analysis were performed. Cell cycle analysis based on propidium iodide (PI) confirmed a decrease in CCRF-CEM cell proliferation, indicating a significant decrease in G1 phase cells from 45.2% for SCR to 40.3% for TET1sh, and an increase in G2/M phase cells from 28.1% for SCR to 37.3% for TET1sh (Fig. 4d). In parallel, we quantified cell death by Annexin V and PI staining followed by flow cytometric analysis (Fig. 4e). While we did not detect significant changes in the fraction of necrotic cells, we found a small decrease in apoptotic cells for TET1sh. We furthermore validated our findings in mouse T-ALL cells, where *TET1* KD using a different shRNA reduced tumor cell proliferation through cell cycle arrest (Additional file 2: Fig. S2). We conclude that loss of *TET1* expression in both mouse and human T-ALL cells leads to reduced cell proliferation primarily through cell cycle arrest mechanisms.

### Knockdown of *TET1* in human T-ALL cells alters DNA (hydroxy)methylation patterns and gene expression programs

**Fig. 5:**
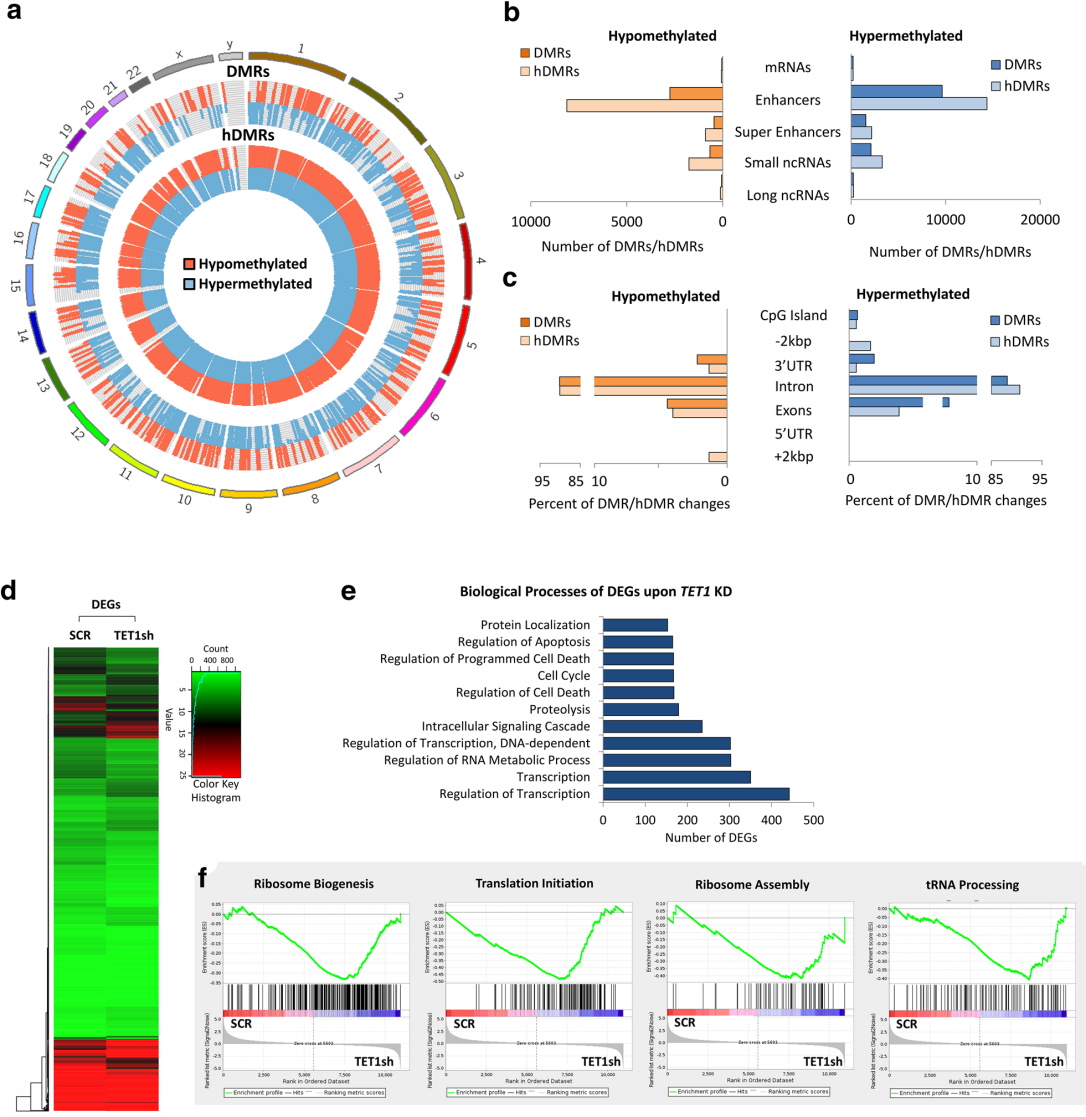

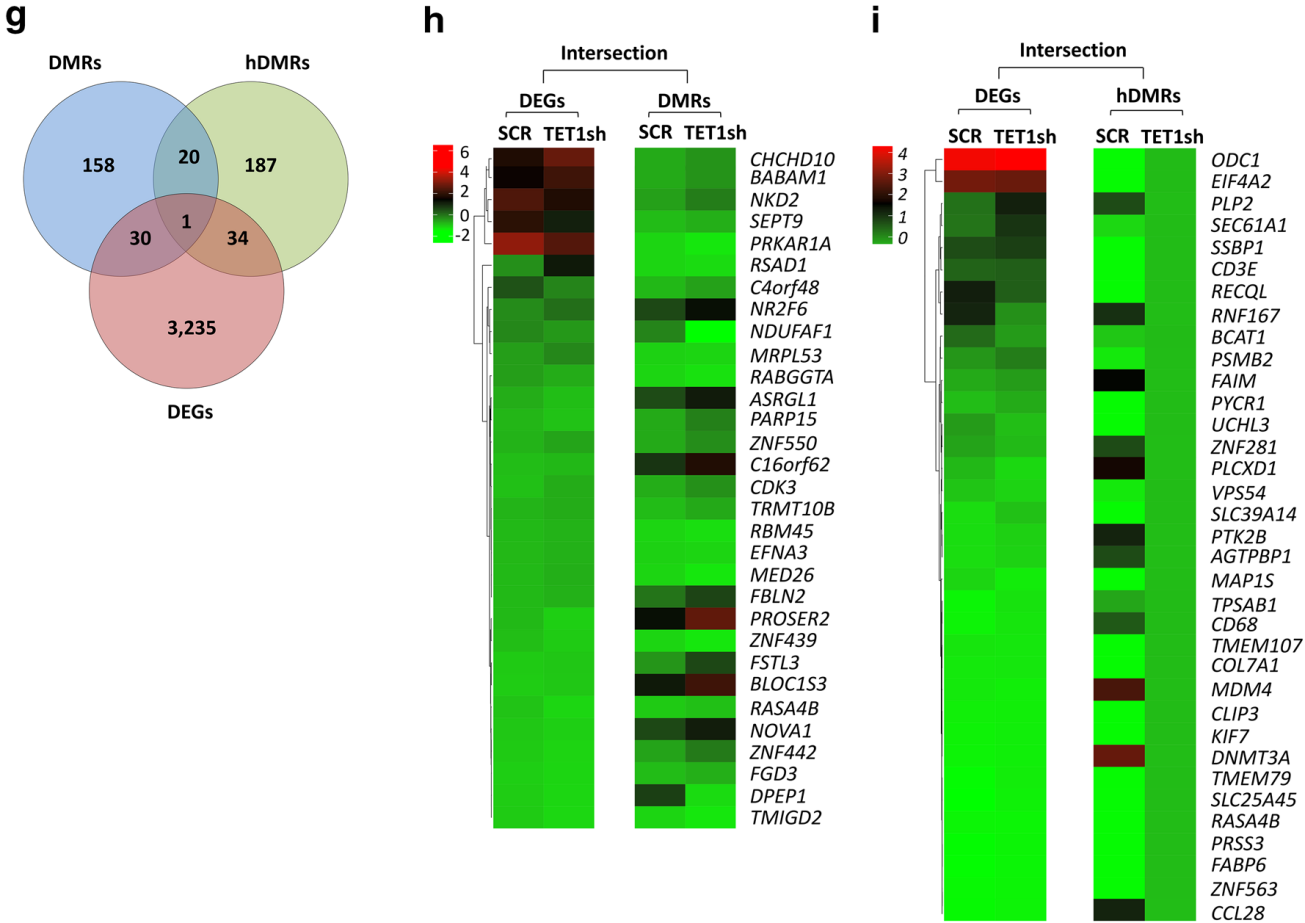
*TET1* KD in human T-ALL cells alters gene expression by changing DNA (hydroxy)methylation. MeDIP-, hMeDIP-, and RNA-seq analysis of human T-ALL cells (CCRF-CEM) before (SCR) and upon *TET1* KD (TET1sh). **a** Genomic distribution of DMRs and hDMRs is displayed as chromosome-based circular plot. Cutoff: log2FC ≥ 1 with a *P* value of ≤ 10^−4^. **b** Hypo- or hypermethylated DMRs and hDMRs are shown annotated for their association with mRNAs, enhancers, super-enhancers, small ncRNAs, and long ncRNAs. **c** Hypo- or hypermethylated DMRs and hDMRs associated with mRNAs are shown annotated for *cis*-regulatory elements: CpG islands, exons, introns, 5′-/3′-UTRs, and sequences greater than 2 kbp upstream or downstream of the nearest gene. **d** RNA-seq analysis: heatmap showing hierarchical clustering of 3302 DEGs. Gene names are listed in Additional file 3. **e** Gene ontology analysis (DAVID) based on DEGs. **f** Gene set enrichment analysis (GSEA) of RNA expression associated with biological processes. **g** Intersection between DMRs, hDMRs, and DEGs. Gene names are listed in Additional file 3. Heatmap showing hierarchical clustering of genes both differentially expressed and differentially (hydroxy)methylated: **h** DEGs and DMRs and **i** DEGs and hDMRs

To investigate the molecular mechanisms underlying reduced tumor cell proliferation upon *TET1* KD in human T-ALL cells, we measured DNA (hydroxy)methylation and RNA expression changes, using MeDIP-, hMeDIP-, and RNA-seq analysis, comparing CCRF-CEM cells before (SCR) and upon *TET1* KD (TET1sh) (Fig. 5). For each sample, we generated 30–40 million Illumina sequencing reads, of which ~ 40–85% were successfully mapped to either strand of the human genome (hg19). To identify statistically significant DMRs and hDMRs, we used a cutoff of log2FC ≥ 1 with a *P* value of ≤ 10^−4^.

We identified a total of 17,712 DMRs and 31,253 hDMRs that increased or decreased significantly upon *TET1* KD in CCRF-CEM cells. The chromosome-based circular plot reveals genome-wide changes in the 5mC and 5hmC patterns upon loss of *TET1* expression (Fig. 5a). Annotating DMRs and hDMRs (both hypo- and hypermethylated) with the nearest gene, we found 259 and 277 DMRs and hDMRs associated with mRNAs, 12,386 and 22,786 with enhancers, 2011 and 3068 with super-enhancers, 2770 and 5054 with small ncRNAs, and 295 and 378 with long ncRNAs, respectively (Fig. 5b). By further annotating DMRs and hDMRs associated with protein-coding genes with *cis*-regulatory elements, we found 3.23% and 2.76% of the DMRs and hDMRs associated with CpG islands, 91.58% and 93.40% with introns, 6.04% and 3.91% with exons, 2.05% and 0.93% with 5′- or 3′-UTRs, and 0% and 1.49% with sequences 2 kbp upstream or downstream of the nearest gene, respectively (Fig. 5c). Thus, *TET1* KD in human T-ALL cells leads to genome-wide changes in DNA (hydroxy)methylation patterns associated mostly with introns.

To determine the effect of alterations in DNA (hydroxy)methylation upon *TET1* KD on RNA expression in CCRF-CEM cells, we next performed RNA-seq. We identified 3300 statistically significant DEGs (Fig. 5d). Of those, 1806 decreased, while 1494 increased in expression upon *TET1* KD. To determine the biological processes associated with the DEGs, we performed gene ontology analysis using DAVID. We found a wide variety of processes including regulation of transcription, RNA metabolism, intracellular signaling as well as cell cycle and cell death-related pathways to be affected (Fig. 5e and Additional file 3). To further associate RNA expression with biological processes, we performed gene set enrichment analysis (GSEA). Among the largest enrichments, we found ribosome biogenesis (*n* = 272), ribosome assembly (*n* = 60), translational initiation (*n* = 136), and tRNA processing (*n* = 81), all associated with protein synthesis (Fig. 5f). Together, these results indicate that reduced proliferation of T-ALL cells upon loss of TET1 function is associated with decreased global protein synthesis. Similarly, inactivation of MYC in T-ALL has previously been shown to shut down global protein synthesis eliciting cellular senescence and tumor regression [16, 34].

To further determine the relationship between DNA (hydroxy)methylation and gene expression changes in CCRF-CEM cells upon *TET1* KD, we selected for DEGs that are associated with changes in 5mC or 5hmC enrichment. We plotted DMRs, hDMRs, and DEGs in a Venn diagram (Fig. 5g). The intersection graph shows a total of 3300 DEGs, as well as 209 DMRs and 242 hDMRs that are associated with protein coding genes. Of the 3300 DEGs, we identified 31 to be associated with DMRs and 35 with hDMRs (Fig. 5h, i). We found that 21 DMRs and hDMRs overlap with each other. One gene, *RASA4B* (RAS P21 Protein Activator 4B), was significantly changed in all three categories, DEGs, DMRs, and hDMRs. We conclude that in T-ALL, MYC-driven overexpression of *TET1* contributes to tumor cell-specific 5mC and 5hmC patterns and thus gene expression programs that are important for enhanced global protein synthesis and tumor cell proliferation.

### Ectopic expression of *TET2* decreased tumor cell proliferation of human T-ALL cells

**Fig. 6:**
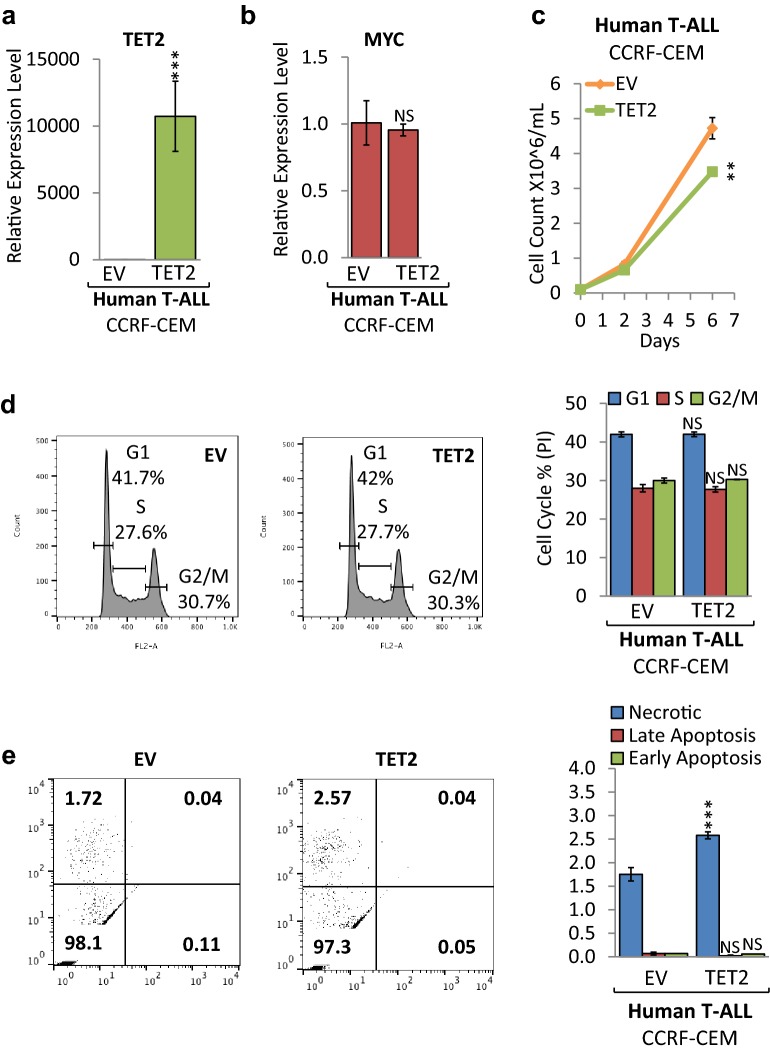
Ectopic *TET2* expression reduces cell proliferation of human T-ALL. Human T-ALL cells (CCRF-CEM), engineered to ectopically express *TET2* (TET2), were compared with empty vector (EV) controls. RT-qPCR analysis of **a** *TET2* and **b** *MYC* expression. RT-qPCR data were normalized to *RPL13a*. **c** Growth curve comparing viable cell counts. **d** Flow cytometric cell cycle analysis based on propidium iodide (PI) staining. Cell cycle distribution (G1, S, and G2/M) displayed in percent. **e** Flow cytometric analysis of apoptosis based on Annexin V/PI staining. Flow cytometry profile of Annexin V staining (*X* axis) and PI (*Y* axis) is shown for representative samples. The lower right quadrant indicates the percentage of early apoptotic cells in each condition; the upper right quadrant indicates the percentage of late apoptotic cells; the upper left quadrant indicates the percentage of necrotic cells. Apoptotic cells (Annexin V-positive cells) are displayed as the percentage of gated cells. Error bars represent mean ± SEM; *n* = 3; two-tailed Student’s *t* test: *P < 0.05; **P < 0.01; ***P < 0.001

To determine whether reconstitution of *TET2* expression in human T-ALL cells affects tumor cell proliferation and viability, we retrovirally introduced full-length TET2 cDNA into CCRF-CEM cells (Fig. 6). RT-qPCR analysis confirmed ectopic *TET2* mRNA expression in CCRF-CEM (TET2) compared to empty vector (EV) control cells (Fig. 6a), while *MYC* expression levels remained unchanged (Fig. 6b). The corresponding growth curve revealed that ectopic *TET2* expression significantly decreases proliferation of CCRF-CEM cells (Fig. 6c). To determine the cellular mechanism underlying reduced cell proliferation, we performed flow cytometric cell viability and cell cycle analysis. Cell cycle analysis based on propidium iodide (PI) indicated no significant change in G1, S, or G2/M phase of the cell cycle (Fig. 6d). In parallel, we quantified cell death by Annexin V and PI staining followed by flow cytometric analysis (Fig. 6e). While we did not detect significant changes in the fraction of apoptotic cells, we found a significant increase in necrotic cells (from 1.7% to 2.6%) upon ectopic *TET2* expression. We conclude that ectopic *TET2* expression in human T-ALL disrupts tumor cell growth and results in reduced cell proliferation primarily through necrosis.

### Ectopic expression of *TET2* in human T-ALL cells alters gene expression programs by changing DNA (hydroxy)methylation

**Fig. 7:**
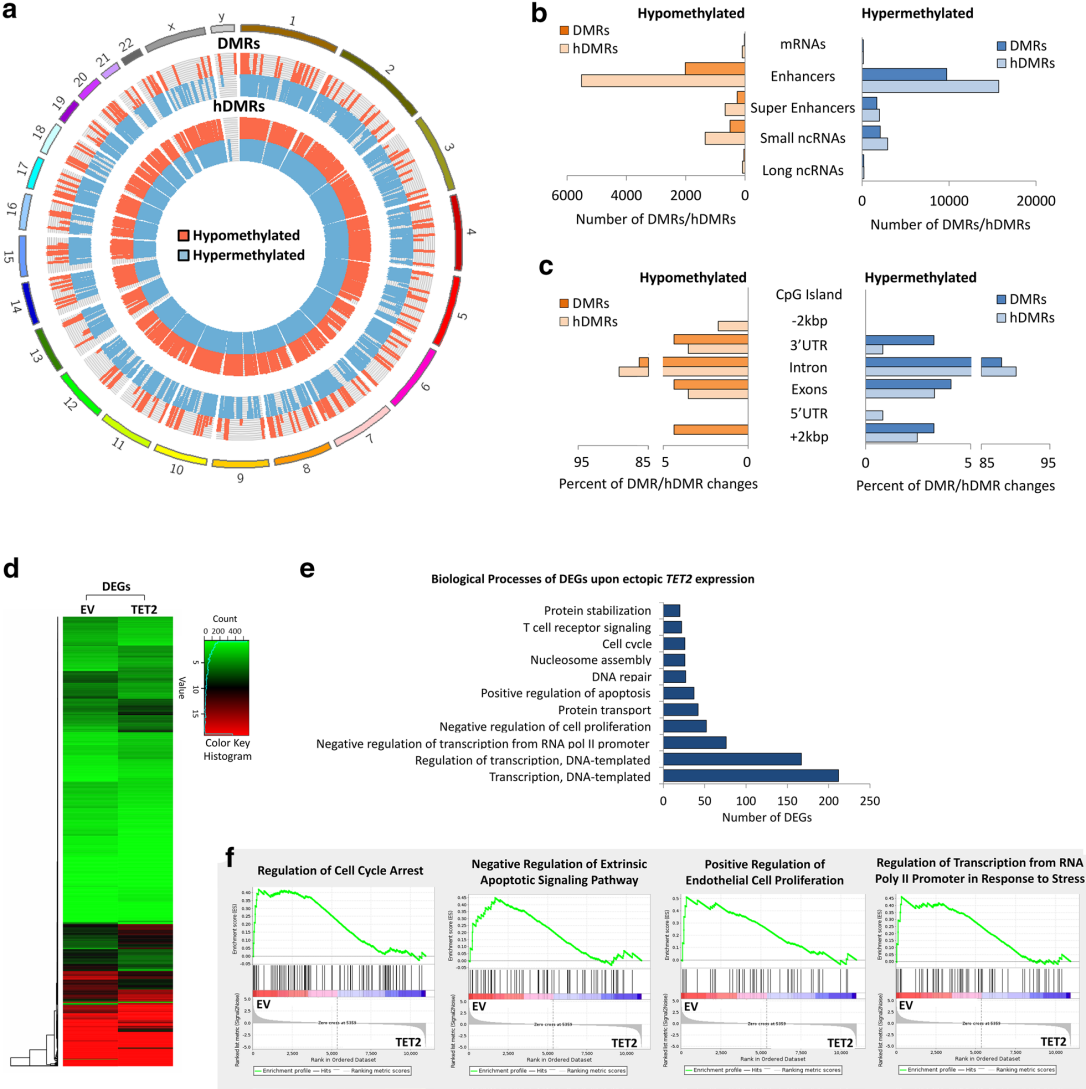

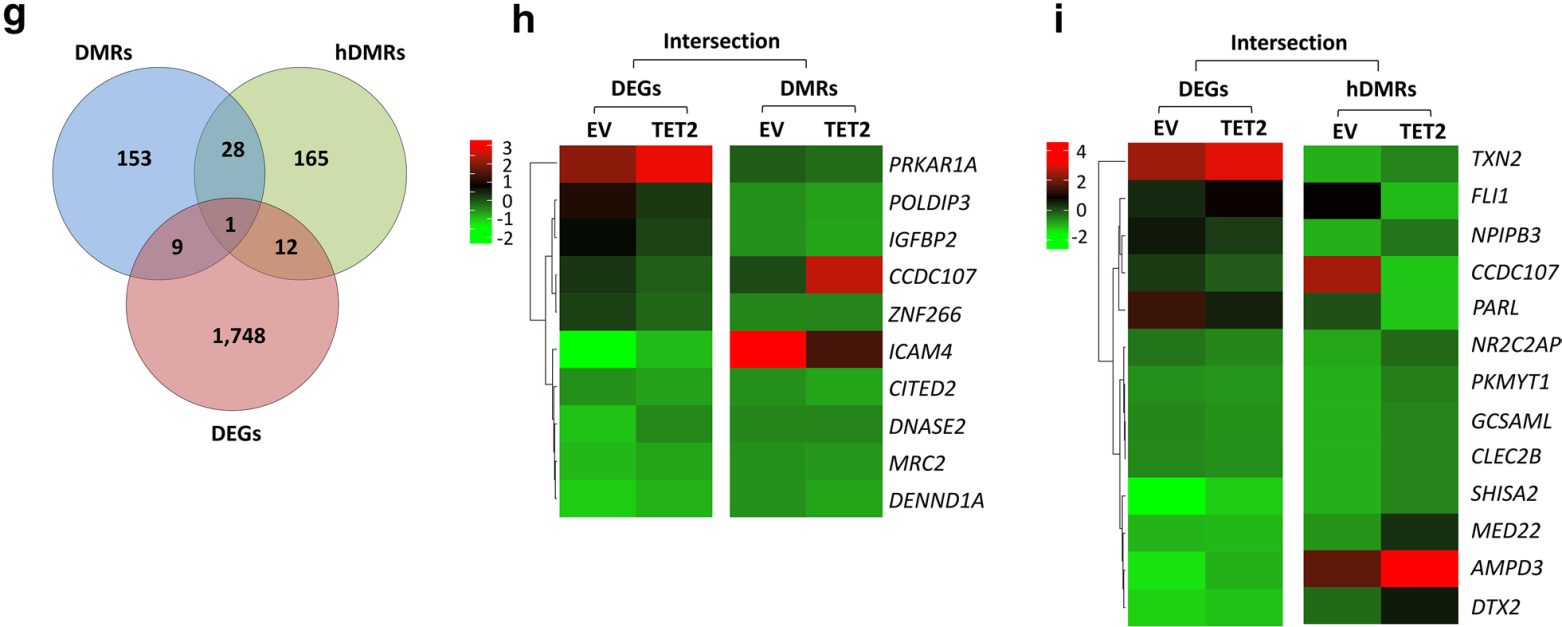
Ectopic *TET2 *expression in human T-ALL cells alters gene expression by changing 5mC and 5hmC patterns. MeDIP-, hMeDIP-, and RNA-seq analysis of human T-ALL cells (CCRF-CEM) upon ectopic expression of *TET2 *(TET2) compared to empty vector (EV) control. **a** Genomic distribution of DMRs and hDMRs is displayed as chromosome-based circular plot. Cutoff: log2FC ≥ 1 with a *P* value of ≤ 10^−4^. **b** Hypo- or hypermethylated DMRs and hDMRs are shown annotated for their association with mRNAs, enhancers, super-enhancers, small ncRNAs, and long ncRNAs. **c** Hypo- or hypermethylated DMRs and hDMRs associated with mRNAs are shown annotated for *cis*-regulatory elements: CpG islands, exons, introns, 5′-/3′-UTRs, and sequences  greater than 2 kbp upstream or downstream of the nearest gene. **d** RNA-seq analysis: heatmap showing hierarchical clustering of 1771 DEGs. Gene names are listed in Additional file 3. **e** Gene ontology analysis (DAVID) based on DEGs. **f** Gene set enrichment analysis (GSEA) of RNA expression associated with biological processes. **g** Intersection between DMRs, hDMRs, and DEGs. Gene names are listed in Additional file 3. Heatmap showing hierarchical clustering of genes both differentially expressed and differentially (hydroxy)methylated: **h** DEGs and DMRs and **i** DEGs and hDMRs

To determine the effect ectopic *TET2* expression has on DNA (hydroxy)methylation and RNA expression in human T-ALL cells, we carried out MeDIP-, hMeDIP-, and RNA-seq analysis, comparing CCRF-CEM cells before (EV) and upon ectopic expression of *TET2* using cDNA (TET2) (Fig. 7). For each MeDIP/hMeDIP sample, 30–40 million Illumina sequencing reads were generated, of which ~ 40–85% were successfully mapped to either strand of the human genome (hg19). To identify DMRs and hDMRs, we performed a genome-wide, unbiased detection by complete tiling of the human genome using a (hydroxy)methylation difference cutoff of log2FC ≥ 1 with a *P* value of ≤ 10^−4^.

We identified a total of 16,666 DMRs and 28,681 hDMRs that were significantly hypo- or hypermethylated upon ectopic *TET2* expression in CCRF-CEM cells. The genomic location of DMRs and hDMRs indicates genome-wide changes in 5mC and 5hmC distribution upon ectopic *TET2* expression (Fig. 7a). Annotating DMRs and hDMRs (hypo- and hypermethylated) with the nearest gene, we found 158 and 238 DMRs and hDMRs associated with mRNAs, 11,746 and 21,224 with enhancers, 1957 and 2665 with super-enhancers, 2588 and 4272 with small ncRNAs, and 226 and 292 with long ncRNAs, respectively (Fig. 7b). We further annotated DMRs and hDMRs associated with mRNA genes with cis-elements. Of the total DMRs and hDMRs, we found 86.96% and 91.93% associated with introns, 4.21% and 3.39% with exons, 3.80% and 2.57% with 5′- or 3′-UTRs, and 3.80% and 2.11% with sequences 2 kbp or more upstream or downstream of the nearest gene, respectively (Fig. 7c). Thus, similar to *TET1* KD, ectopic expression of *TET2* in human T-ALL cells led to genome-wide changes in 5mC and 5hmC patterns associated mostly with intron sequences.

To determine the effect of alterations in DNA (hydroxy)methylation upon ectopic *TET2* expression on RNA expression in CCRF-CEM cells, we next performed RNA-seq. We identified 1771 statistically significant DEGs, of which 917 genes decreased, while 854 increased in expression upon ectopic *TET2* expression (Fig. 7d). To determine the biological processes associated with the DEGs, we performed gene ontology analysis using DAVID (Fig. 7e). We found regulation of transcription, negative regulation of cell proliferation, and positive regulation of apoptosis among the processes associated with ectopic *TET2* expression in T-ALL. We further performed gene enrichment analysis to associate RNA expression with biological processes. The results indicate that upon ectopic *TET2* expression, there is a decreasing trend for genes involved in cell cycle arrest (*n* = 84), negative regulation of extrinsic apoptotic signaling (*n* = 66), positive regulation of endothelial cell proliferation (*n* = 77), and regulation of transcription from RNA Pol II promoter in response to stress (*n* = 58) (Fig. 7f).

To determine the relationship between DNA (hydroxy)methylation and gene expression changes in CCRF-CEM cells upon ectopic *TET2* expression, we selected for DEGs that are associated with changes in 5mC or 5hmC enrichment. We plotted DMRs and hDMRs associated with mRNAs, as well as DEGs in a Venn diagram (Fig. 7g). The intersection graph shows a total of 1771 DEGs, as well as 150 DMRs and 200 hDMRs that are associated with protein-coding genes. Of the 1771 DEGs, we identified 10 to be associated with DMRs and 13 with hDMRs (Fig. 7h, i). We found that 25 DMR- and hDMR-associated protein-coding genes overlap. *CCDC107* (coiled-coil domain-containing protein 107), was significantly changed in all three categories: DEGs, DMRs, and hDMRs. Taken together, we conclude that ectopic expression of *TET2* in T-ALL alters 5mC and 5hmC patterns and thus gene expression programs resulting in reduced tumor cell proliferation.

### TET1, TET2, and MYC target genes overlap

**Fig. 8:**
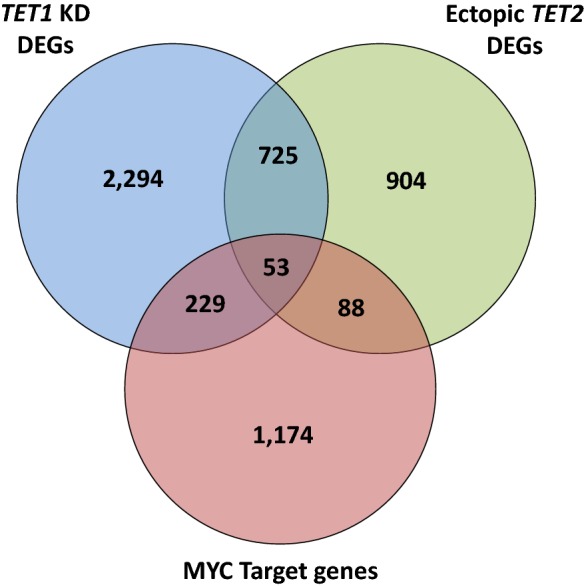
TET1, TET2, and MYC target genes overlap. Overlap between DEGs upon *TET1* KD and ectopic *TET2* expression in human T-ALL cells (CCRF-CEM), and previously identified MYC target genes, obtained from the Molecular Signatures Database (MSigDB) [35–37]. The intersection between genes is displayed as Venn diagram. Gene names are listed in Additional file 3

To determine the overlap between TET1, TET2, and MYC target genes in T-ALL, we used the DEGs upon *TET1* KD and ectopic expression of *TET2* in human T-ALL cells (CCRF-CEM) and compared them with previously identified MYC target genes [35–37] (Fig. 8). Of the 3300 DEGs identified upon *TET1* KD and the 1771 DEGs identified upon ectopic expression of *TET2*, we found 778 genes overlap with each other. At the same time, we found 282 genes that were both regulated by TET1 and MYC, and 141 genes that were both regulated between TET2 and MYC. Lastly, we found 53 genes that all three data sets had in common. Taken together, this indicates that there is some overlap between TET1 and TET2 targets, even though the two enzymes have distinct functions in T-ALL. At the same time, fewer TET1 and TET2 targets overlap with MYC, revealing that the majority of TET targets are not directly occupied by MYC, but rather regulated indirectly highlighting an indirect genome-wide mechanism.

## Discussion

This study demonstrates for the first time that the MYC oncogene deregulates the expression of TET methylcytosine dioxygenases and thereby global DNA (hydroxy)methylation and gene expression programs to maintain tumor cell proliferation. While aberrant DNA methylation is a characteristic feature of tumor cells, the mechanisms of how tumor cell-specific DNA (hydroxy)methylation patterns are written, maintained, and erased through the coordinated action of DNA methylating and demethylating enzymes are poorly understood. Here, we reveal a novel mechanism through which MYC establishes and maintains tumor cell-specific DNA (hydroxy)methylation and gene expression programs in a genome-wide fashion.

By carrying out a comprehensive genome-wide DNA (hydroxy)methylation analysis, we show that cellular senescence and tumor regression upon MYC inactivation in a mouse model of T-ALL (*EµSRα*-*tTAα;tet*-*o*-*MYC*) is associated with genome-wide changes in 5mC and 5hmC patterns. Using this T-ALL model, we have previously reported that tumor regression depends on activation of cellular senescence pathways associated with genome-wide changes in chromatin structure including histone acetylation and methylation, associated with heterochromatin formation [3, 18, 30]. Together, our MeDIP- and hMeDIP-seq analyses reveal genome-wide changes in 5mC and 5hmC patterns associated with a wide variety of biological processes upon MYC inactivation, cellular senescence, and tumor regression, indicating that MYC maintains tumor cell-specific DNA (hydroxy)methylation patterns in T-ALL.

Shedding light on the underlying molecular mechanism, we found that in T-ALL, *TET1* is overexpressed, while *TET2* transcription is repressed in a MYC-dependent fashion across all human T-ALL cell lines and clinical specimens we analyzed. Consistently, we were able to demonstrate that MYC inactivation in T-ALL arising in *EµSRα*-*tTA;tet*-*o*-*MYC* mice leads to an inverse expression pattern, decreasing *TET1* levels, while increasing *TET2* levels. Together with the ChIP data indicating that MYC binds to the *TET1* and *TET2* loci, our results implicate a direct transcriptional regulation. However, we cannot exclude that, besides being controlled by MYC directly, *TET1* and *TET2* expression might also be regulated indirectly through changes in other MYC target genes. Both DNMT and TET expression and activity have been found deregulated in various cancer types, including hematologic malignancies [38–40]. We conclude that the MYC oncogene upregulates *TET1* while suppressing *TET2* expression in T-ALL, and speculated whether TET function was essential for tumor maintenance. Similarly, we previously reported that in T-ALL and Burkitt lymphoma MYC directly controls the overexpression of *DNMT3B* for tumor maintenance, maintaining specific 5mC and thus gene expression patterns [26].

Indeed, we found that *TET1* KD or ectopic expression of *TET2* decreased cell proliferation in T-ALL and was associated with genome-wide changes in 5mC and 5hmC, suggesting a tumor promoting function of TET1, and a tumor suppressing role for TET2. Our findings are consistent with recent reports that TET1 acts as an oncogene in acute myeloid leukemia (AML) development and that high *TET1* levels are predictive of poor overall survival in AML [41]. Oncogenic TET1 plays an important role in the development of MLL-rearranged leukemia [42–44]. *TET1* is also overexpressed in 40% of patients with triple-negative breast cancer, where it is associated with DNA hypomethylation and activation of oncogenic pathways, leading to poor overall survival [45]. However, *TET1* expression has been found low in many other solid tumor types including colon, gastric, and ER-negative breast cancer [46–49], where *TET1* is downregulated through miR-29, HMGA2, or NF-ĸB activation [50, 51]. Thus, TET1 may act as either tumor promotor or suppressor dependent on the context, a notion that also has been reported for other epigenetic regulators, including DNMTs.

In T-ALL, we found loss of TET1 function to be associated with decreased ribosome biogenesis and assembly, translational regulation, and tRNA processing. Indeed, MYC is well known to regulate ribosome biogenesis and translation through multiple mechanisms (reviewed in [52]), and we previously demonstrated that MYC inactivation leads to shutdown of global protein synthesis resulting in cellular senescence and tumor regression [16, 34]. Our finding that in T-ALL loss of *TET1* expression is linked to a decrease in ribosome biogenesis and translational regulation might provide a novel mechanism of how MYC regulates protein synthesis through alterations in DNA (hydroxy)methylation patterns. Consistent with our findings in T-ALL, high *TET1* expression also correlated with upregulation of RNA transport and ribosome biogenesis pathways in AML [41]. In contrast to TET1, our results show that TET2 acts as a tumor suppressor in T-ALL. We found ectopic expressing of *TET2* to be associated with a decrease in cell cycle regulation, negative regulation of apoptosis, and positive regulation of cell proliferation. While the absence of TET2 in MYC-driven tumors does not automatically mean it reinforces the tumor state, our ectopic *TET2* expression experiments indicate a role as tumor suppressor. Similarly, TET2 has been reported to be a tumor suppressor in B-cell lymphomagenesis [53].

*TET1* KD and ectopic *TET2* expression in T-ALL causes genome-wide changes in both 5mC and 5hmC patterns. *TET1* KD in T-ALL causes differential hydroxymethylation at *KLF4*, critical for regulation of proliferation, differentiation, apoptosis, and somatic cell reprogramming [54]. There are several TET2 targets in T-ALL that overlap with AML, such as *CCDC84*, *CHKA*, *GNGT2*, *GPS2*, *IL20RB*, *LIN7B*, *PPAN*, *PROCA1*, *RECQL4*, *TMSB10*, *CHKB* (fusion), and *PABPN1* (fusion). It is noteworthy that restoration of TET2 in AML promotes DNA demethylation, cell differentiation, and cell death, leading to a block in self-renewal of hematopoietic stem cells, causing myeloid differentiation [55].

**Fig. 9:**
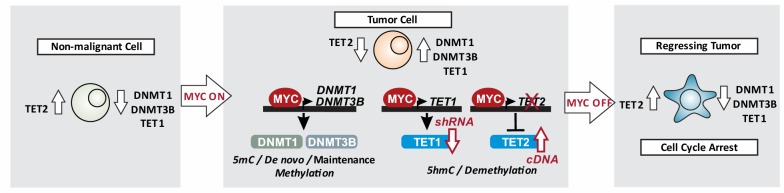
Working model: The MYC oncogene controls *DNMT1* and *DNMT3B*, as well as *TET1* and *TET2* expression in T-ALL. Non-malignant cells: *MYC* levels are low, corresponding with low *TET1*, as well as *DNMT1* and *DNMT3B* expression. Tumor cells: *MYC* levels are constitutively high, driving the expression of *TET1* as well as *DNMT1* and *DNMT3B*, while suppressing *TET2*. Regressing tumor: *MYC* inactivation in T-ALL causes tumor regression through cellular senescence. This is associated with diminished *DNMT1*, *DNMT3B*, and *TET1* levels, while *TET2* expression is increased. Loss of *TET1* function (shRNA) and reconstitution of *TET2* expression (cDNA) led to broad changes in 5mC and 5hmC patterns, in turn affecting a variety of cellular processes causing reduced tumor cell proliferation. Taken together, MYC induces and maintains a tumor cell-specific global 5mC and 5hmC patterns through control of *DNMT* and *TET* expression

While the exact role of TET1 and TET2 in regulating DNA (hydroxy)methylation outside of developmental processes is not well understood, our findings indicate distinct functions of TET1 and TET2 in MYC-driven tumor maintenance. In our working model (Fig. 9), the MYC oncogene directly drives the transcription of *TET1* (as well as *DNMT1* and *DNMT3B* [26]), while suppressing *TET2*. Inactivation of MYC inverts the expression pattern of both DNMTs and TETs, eliciting cellular senescence and tumor regression. Loss of *TET1* and overexpression of *TET2* cause genome-wide changes in 5mC and 5hmC patterns. We found a majority of changes in DNA (hydroxy)methylation patterns occurred in intron sequences. These observations are consistent with other studies, indicating that TET1 localizes to gene bodies and introns of a large number of genes [56–58]. Our data suggest that TET1 and TET2 have distinct sets of genomic targets in turn affecting distinct cellular processes, despite some overlap in differentially expressed genes. In this regard, it would be of interest to uncover which of the differentially (hydroxy)methylated genes are directly bound by TET1 or TET2 in T-ALL using genome-wide location analysis. Furthermore, it would be interesting to determine whether the genomic targets of DNMT1 and DNMT3B in this context overlap with TET1 or TET2, and whether there is any synergistic effect between the components of the DNA methylating and demethylating enzymes contributing to MYC-driven tumor maintenance.

## Conclusions

This study provides novel evidence that MYC directly deregulates the expression of *TET1* and *TET2* in T-ALL to maintain 5mC and 5hmC patterns in a genome-wide fashion, which is associated with tumor cell-specific gene expression. Our results reveal that the MYC oncogene establishes and maintains tumor cell-specific DNA (hydroxy)methylation patterns on a genome-wide level by modulating the expression of individual components of the DNA methylating and demethylating machinery. Our working model indicates a coordinated interplay between the components of the DNA methylating and demethylating machinery contributing to MYC-driven tumor maintenance, highlighting the potential of specific TET enzymes for therapeutic strategies. Targeting DNMTs or TET enzymes pharmacologically for therapeutic anticancer strategies may be a promising concept, even though specific small molecule inhibitors are currently still elusive.

## Methods

### Cell culture and treatment

Mouse T-ALL cell lines, derived from the transgenic T-ALL mouse model (*EµSRα*-*tTAα;tet*-*o*-*MYC*), were kindly provided by Dr. Dean W. Felsher, Stanford University [5]. Human Burkitt lymphoma-like cells (P493-6) [59], T cell leukemia/lymphoma cell lines (JURKAT, CCRF-CEM, MOLT3, MOLT4, and CCRF-HSB-2), and adherent packaging cell lines (HEK293T and Phoenix-ampho) were obtained from American Type Culture Collection (ATCC). To turn off expression of the tetracycline-regulated MYC in the mouse model and in P493-6 cells, 20 ng/mL doxycycline (DOX) was added to the cell culture medium for the indicated times. All leukemia/lymphoma cell lines were passaged less than 8 times, maintained in RPMI1640 supplemented with 10% FBS, 1% penicillin/streptomycin, 1% l-glutamine, and 50 μM 2-mercaptoethanol, and incubated at 37 °C humidified with 5% CO_2_. Adherent cell lines were passaged less than 8 times, maintained in DMEM supplemented with 10% FBS, 1% penicillin/streptomycin, 1% l-glutamine, and 50 μM 2-mercaptoethanol, and incubated at 37 °C humidified with 5% CO_2_. Routine mycoplasma detection is done on all cell lines to eliminate risk of contamination.

### shRNA-mediated knockdown

Cell lines were infected with lentiviral vectors (pLKO.1-puro) containing either scrambled control (SCR) or specific shRNA directed against *TET1*. The specific oligo sequences of shRNA are: Hs. TET1sh CCGGACACAACTTGCTTCGATAATTCTCGAGAATTATCGA AGCAAGTTGTGTTTTTTG and Mm. TET1sh CCGGCAACTTGCATCCACGATTAATCTCGAGATTAATCGTGGATGCAAGTTGTTTTTG. Briefly, HEK293T cells were transfected using Lipofectamine 2000 (Invitrogen) with pLKO.1-puro, pPAX2, and pMD.G plasmids. Virus particles were collected for spinoculation at 2400 rpm for 2 h at 32 °C. Upon selection of positive cells with 2–4 µg of puromycin, knockdown of *TET1* was confirmed by RT-qPCR.

### Ectopic gene expression

Cell lines were infected with retroviral vectors (pMSCV-PIG) containing either empty vector (EV) or a mouse TET2 full-length cDNA. pcDNA3-Tet2 (#60939) and MSCV PIG (Puro-IRES GFP) (#18751) were obtained from Addgene. Mm TET2 cDNA was subcloned from pCDNA3-Tet2 using SnaBI/NotI restriction sites, into adapted pMSCV-PIG (Puro-IRES-GFP) plasmid using HpaI/NotI restriction sites. Adapted pMSCV-PIG plasmid vector was modified with destroyed second EcoRI site and introduced NotI site and was a gift from Dr. Honglin Li, Augusta University. pMSCV-PIG-MmTET2 clone was verified via DNA sequencing. Briefly, Phoenix-Ampho cells were transfected using Lipofectamine 2000 (Invitrogen) with pMSCV-PIG plasmid. Virus particles were collected for spinoculation at 2400 rpm for 2 h at 32 °C. Upon selection of positive cells with 2–4 µg of puromycin, cDNA expression was detected using RT-qPCR for *TET2*.

### Tissue collection

Human spleen (total RNA) obtained from a healthy donor was purchased from Zyagen Inc. Human PBMCs (total RNA) were obtained from the Augusta University Biorepository.

### RNA extraction and analysis of gene expression

Total RNA was isolated using the NucleoSpin RNA Kit including DNase-I digest (Machery-Nagel Inc.) following the manufacturer’s protocol. 0.5 μg RNA was reverse transcribed into cDNA using the iScript cDNA Kit (BioRad). Quantitative PCR (qPCR) was performed using iTAQ Universal SYBR GREEN (BioRad) in an ABI StepOne Plus analyzer (Applied Biosystems). Specific primer sequences are as follows: *Mm MYC* F: TCTCCATCCTATGTTGCGGTC, R: TCCAAGTAACTCGGTCATCATCT; *Mm ODC1* F: GACGAGTTTGACTGCCACATC, R: CGCAACATAGAACGCATCCTT; *Mm TET1* F: ATTTCCGCATCTGGGAACCTG, R: GGAAGTTGATCTTTGGGGCAAT; *Mm TET2* F: TGCTTTCCCAACACGGAACTA, R: GCACCATTAGGCATTAGCACAAT; *Mm TET3* F: TGCGATTGTGTCGAACAAATAGT, R: TCCATACCGATCCTCCATGAG; *Mm UBC* F: AGCCCAGTGTTACCACCAAG, R: ACCCAAGAACAAGCACAAGG; *Hs MYC* F: CTGCGACGAGGAGGAGAA, R: GGCAGCAGCTCGAATTTCTT; *Hs TET1* F: CATCAGTCAAGACTTTAAGCCCT, R: CGGGTGGTTTAGGTTCTGTTT; *Hs TET2* F: GATAGAACCAACCATGTTGAGGG, R: TGGAGCTTTGTAGCCAGAGGT; *Hs TET3* F: TCCAGCAACTCCTAGAACTGAG, R: AGGCCGCTTGAATACTGACTG; *Hs RPL13A* F: CGGACCGTGCGAGGTAT, R: CACCATCCGCTTTTTCTTGTC.

## RNA sequencing

Total RNA was extracted as described above, and samples were quantified using Nanodrop and qualified by agarose gel electrophoresis. Briefly, mRNA was isolated from total RNA with NEBNext PolyA mRNA Magnetic Isolation Module. Alternatively, rRNA was removed from the total RNA with a RiboZero Magnetic Gold Kit. The enriched mRNA or rRNA-depleted RNA was used for RNA-seq library preparation using KAPA Stranded RNA-Seq Library Prep Kit (Illumina). The completed libraries were qualified on Agilent 2100 Bioanalyzer for concentration, fragment size distribution between 400 and 600 bp, and adapter dimer contamination. The DNA fragments in mixed libraries were denatured with 0.1 M NaOH to generate single-stranded DNA molecules, loaded onto channels of the flow cell at 8 pM concentration, and amplified in situ using TruSeq SRCluster Kit v3-cBot-HS (#GD-401-3001, Illumina). Sequencing was carried out by running 150 cycles with paired-end reads, using the Illumina HiSeq 4000 according to the manufacturer’s instructions. RNA sequencing was performed by Arraystar Inc. (Rockville, MD).

## RNA-seq data analysis

After quality control, the fragments were 5′,3′-adaptor-trimmed and filtered ≤ 20 bp reads with cutadapt software. The trimmed reads were aligned to reference genome with Hisat 2 software [60]. The expression level (FPKM value) of known genes and transcripts were calculated using ballgown through the transcript abundances estimated with StringTie [61, 62]. The number of identified genes and transcripts per group was calculated based on the mean of FPKM in group ≥ 0.5. Principal component analysis (PCA), correlation analysis, hierarchical clustering, gene ontology (GO), pathway analysis, scatter plots, and volcano plots are performed for the differentially expressed genes in R or Python environment for statistical computing and graphics. RNA sequencing data analysis was performed by Arraystar Inc. (Rockville, MD). Data sets are deposited in GEO under accession number GSE126029.

### Cell cycle analysis using propidium iodide

Cells were fixed in 70% methanol at − 20 °C for a minimum of 72 h and stained using a propidium iodide (PI) solution containing PBS + 0.5% BSA, 50 µg/mL PI (Acros Organics), and 200 µg/mL RNase A (Thermo Fisher). Cells were then analyzed immediately on a FACScalibur flow cytometer (Becton–Dickinson). FACS data were analyzed using FlowJo software (Tree Star).

### Apoptosis analysis using Annexin V/propidium iodide

Annexin V-FITC and PI staining was used for the study of cell cycle distribution and apoptosis using the Annexin V-FITC Early Apoptosis Detection Kit (Cell Signaling). Briefly, cells were washed in PBS and suspended in 1X Annexin V binding buffer. Annexin V-FITC conjugate and propidium iodide were incubated for 10 min on ice and immediately analyzed on a FACScalibur flow cytometer (Becton–Dickinson). FACS data were analyzed using FlowJo software (Tree Star).

## MeDIP and hMeDIP sequencing

Genomic DNA was isolated using the Nucleospin Tissue Kit with RNase A (Macherey-Nagel Inc.). Purified genomic DNA was sonicated to ~ 200–800 bp fragments, and 1 µg of fragmented DNA was ligated to Illumina’s genomic adapters with Genomic DNA Sample Kit (#FC-102-1002, Illumina), following the manufacturer’s instructions. ~ 300–900 bp ligated DNA fragments were further immunoprecipitated using an anti-5-Methylcytosine antibody (for MeDIP) or an anti-5-Hydroxymethylcytosine antibody (for hMeDIP). The enriched DNA was amplified by PCR and purified by AMPure XP beads. The DNA fragments in mixed libraries were denatured with 0.1 M NaOH to generate single-stranded DNA molecules, loaded onto channels of the flow cell at 8 pM concentration, and amplified in situ using HiSeq 3000/4000 PE Cluster Kit (#PE-410-1001, Illumina). Sequencing was carried out by running 150 cycles with paired-end reads, using HiSeq 3000/4000 SBS Kit (#FC-410-1003, Illumina) on Illumina HiSeq 4000 according to the manufacturer’s instructions. MeDIP and hMeDIP sequencing was performed by Arraystar Inc. (Rockville, MD).

## MeDIP- and hMeDIP-seq data analysis

Raw sequencing data generated from Illumina HiSeq 4000 that pass the Illumina chastity filter are used for following analysis. The fragments were 5′, 3′-adaptor-trimmed and filtered ≤ 16 bp reads with cutadapt software. Trimmed reads (trimmed 5′,3′-adaptor bases) are mapped to reference genome (hg19 or mm10) with Hisat2 software. The mapped reads were used for statistically significant unbiased methylation/hydroxymethylation region detection. LncRNA-, mRNA-, and small ncRNA-associated DMRs- and hDMRs-enriched regions (peaks) with statistically significant were identified by diffReps (cutoff: log2FC ≥ 1, *P* value ≤ 10^−4^) [63]. LncRNA-, mRNA-, and small ncRNA-associated DMRs and hDMRs within promoter were annotated by the nearest gene using the UCSC RefSeq database. Promoter peaks were defined as 2000 bp upstream and downstream from the transcription start site (TSS); gene body peaks were defined as +2000 bp downstream of the transcription start site (TSS) to the transcription termination site (TTS); and intergenic peaks were defined as the other genomic regions not included in either promoters or gene body. MeDIP and hMeDIP analysis was performed by Arraystar Inc. (Rockville, MD). Data sets are deposited in GEO under accession number GSE126029.

## Gene set enrichment analysis (GSEA)

GSEA (version 2.2.4) was performed using Java-based software (http://software.broadinstitute.org/gsea/index.jsp) to distinguish important biological processes pathways enriched between two samples [36]. Enrichment score was calculated for gene sets within a pathway and was selected based on nominal *P* ≤ 0.05.

## Functional annotation of biological processes

Database for Annotation, Visualization and Integrated Discovery (DAVID) (version 6.8) analysis was performed based on online software (https://david.ncifcrf.gov/home.jsp) [64]. Gene ontology (GO) analysis of biological processes was performed to indicate genes expressed differentially in biological pathways between two samples. Statistical parameters were based on default DAVID parameters including a threshold count of 2 with EASE of 0.1, *P* value ≤ 0.05, and Benjamini threshold ≤ 1.

### Clinical specimen data analysis

Gene expression analysis in clinical specimens was performed using Oncomine (http://www.oncomine.org) data sets.

### Statistical analysis

All experiments were performed on biological replicates unless otherwise specified. Sample size is reported in the respective figure legends. All quantitative PCR were run in triplicates, and standard deviation is shown in the figures. Two-tailed unpaired student’s *t* test was used to calculate *P* values; statistically significant values are specified in the figure legends. Statistical significance: NS = not significant *P < 0.05, **P < 0.01, ***P < 0.001.

## Additional files

**Additional file 1: Fig. S1.** MYC inactivation in T-ALL. T-ALL cells (6780) derived from *EµSRα*-*tTAα;tet*-*o*-*MYC* mice were treated with 20 ng/mL DOX for 2 days. RT-qPCR analysis of *MYC*. RT-qPCR data were normalized to *UBC*. Error bars represent mean ± SEM; *n* = 3; two-tailed Student’s *t* test: ***P < 0.001.

**Additional file 2: Fig. S2.** *TET1* knockdown reduces cell proliferation of mouse T-ALL cell lines. Mouse T-ALL cells (*EµSRα*-*tTAα;tet*-*o*-*MYC*) were compared before (SCR) and upon *TET1* KD (TET1sh). RT-qPCR analysis of **a** *MYC* and *TET1*, **b** *TET2* and *TET3*. RT-qPCR data were normalized to *UBC*. **c** Growth curve comparing viable cell counts. **d** Flow cytometric cell cycle analysis based on propidium iodide (PI) staining. The cell cycle distribution (G1, S, and G2/M) is displayed in percent. Error bars represent mean ± SEM; *n* = 3; two-tailed Student’s *t* test: *P < 0.05; **P < 0.01; ***P < 0.001.

**Additional file 3.** Detailed lists of genes used in DMR, hDMR, DEG, GSEA, and intersection analysis. List of genes associated with DMRs and hDMRs in mouse T-ALL cells (*EµSRα-tTAα;tet-o-MYC*) (6780) before and upon 2 days of MYC inactivation (+DOX). List of genes associated with DMRs and hDMRs, as well as DEGs and intersection analysis in human T-ALL (CCRF-CEM) upon *TET1* KD. List of genes associated with DMRs and hDMRs, as well as DEGs and intersection analysis in human T-ALL (CCRF-CEM) upon ectopic* TET2* expression. List of genes from GSEA analysis in human T-ALL (CCRF-CEM) upon *TET1 *KD. List of genes used in GSEA analysis in human T-ALL (CCRF-CEM) upon ectopic *TET2* expression. Gene lists used for intersection analysis of MYC target genes, TET1 DEGs, and TET2 DEGs.

## Data Availability

The data sets generated and/or analyzed during the current study are available in the GEO repository, under accession number GSE126029.
