# Supplementary figures and images for: MYC deregulates TET1 and TET2 expression to control global DNA (hydroxy)methylation and gene expression to maintain a neoplastic phenotype in T-ALL
Source: Epigenetics Chromatin. 2019 Jul 2;12:41. doi: 10.1186/s13072-019-0278-5 (PMC6604319; doi:10.1186/s13072-019-0278-5)

**Figure S1:**

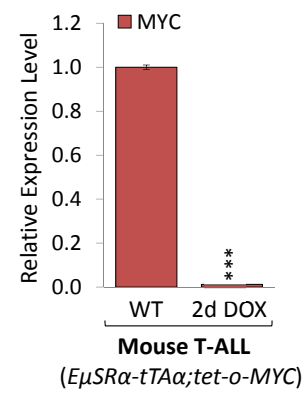

Supplement: Supplementary file 1 — Additional file 1: Fig. S1. MYC inactivation in T-ALL. T-ALL cells (6780) derived from EµSRα-tTAα;tet-o-MYC mice were treated with 20 ng/mL DOX for 2 days. RT-qPCR analysis of MYC. RT-qPCR data were normalized to UBC. Error bars represent mean ± SEM; n = 3; two-tailed Student’s t test: ***P < 0.001. [file 13072_2019_278_MOESM1_ESM.pdf]

**Figure S2:**

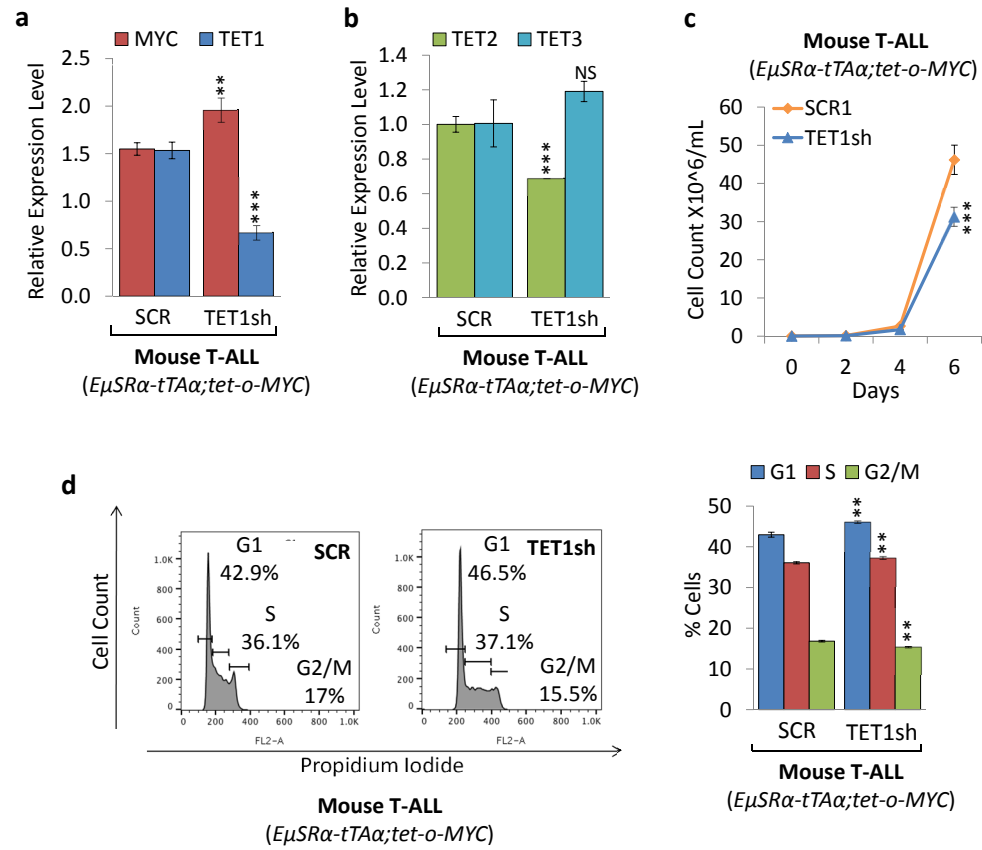

Supplement: Supplementary file 2 — Additional file 2: Fig. S2. TET1 knockdown reduces cell proliferation of mouse T-ALL cell lines. Mouse T-ALL cells (EµSRα-tTAα;tet-o-MYC) were compared before (SCR) and upon TET1 KD (TET1sh). RT-qPCR analysis of a MYC and TET1, b TET2 and TET3. RT-qPCR data were normalized to UBC. c Growth curve comparing viable cell counts. d Flow cytometric cell cycle analysis based on propidium iodide (PI) staining. The cell cycle distribution (G1, S, and G2/M) is displayed in percent. Error bars represent mean ± SEM; n = 3; two-tailed Student’s t test: *P < 0.05; **P < 0.01; ***P < 0.001. [file 13072_2019_278_MOESM2_ESM.pdf]
